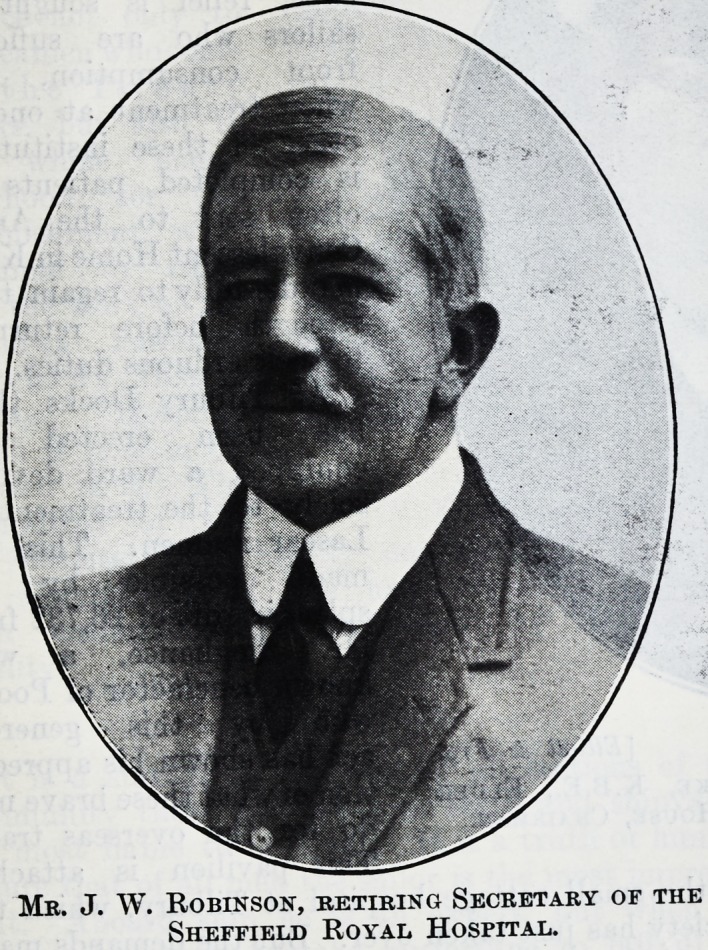# Sheffield Hospital News

**Published:** 1924-07

**Authors:** 


### SHEFFIELD HOSPITAL NEWS.

The resignation of Mr. J. W. Robinson, the Secretary of the Royal Hospital, Sheffield, which we recorded last month, has caused great regret both in the city and in the hospital itself, where he is exceedingly popular with the staff, patients and visitors alike. The secretary's work has, however, become exceedingly onerous, and Mr. Robinson's retirement, as a member of the hospital staff writes, " has not come as a surprise to those who know the indefatigable way in which he has carried out his duties under all conditions. His long and honourable service to the hospital will leave that institution under a very deep debt of gratitude. The Board and medical

Mr. J. W. Robinson, retiring Secretary of the Sheffield Royal Hospital.

staff are deeply sorry to lose the services of such a loyal friend, but they realise the claims of a rest from arduous duties, and they wish him a very hearty God-speed, and hope he will enjoy a well-earned rest. His successor will have a difficult task to fulfil if he is to carry out the ideals that Mr. Robinson has ever had before him, and his advice will always be welcomed by those who are concerned in the administration of the hospital." During his forty-two years' service as Secretary Mr. Robinson has never missed either an annual or a quarterly meeting of governors.

### New Wing at Royal Infirmary.

The new wing of the Sheffield Royal Infirmary for the treatment of nose and throat ailments is about to be opened. The new building will cost £7,600, excluding furnishing, and there will be thirty beds—twenty-four adult and six children. The wing will be equipped with an operating theatre for use by oral cases, and by the out-patients' department for accidents and emergency cases, and there will be consulting and waiting rooms for the oral patients. Nose and throat diseases have increased very much of late in Sheffield, especially among children, and the new department will be a valuable addition to the parent institution.

## Figures and Tables

**Figure f1:**